# Human trophoblast requires galectin-3 for cell migration and invasion

**DOI:** 10.1038/s41598-018-38374-w

**Published:** 2019-02-14

**Authors:** Ž. Bojić-Trbojević, M. Jovanović Krivokuća, A. Vilotić, N. Kolundžić, I. Stefanoska, F. Zetterberg, U. J. Nilsson, H. Leffler, Lj. Vićovac

**Affiliations:** 1 Laboratory for Biology of Reproduction, Institute for the Application of Nuclear Energy, University of Belgrade, Banatska 31b, 11080 Belgrade, Serbia; 2 Galecto Biotech AB, Sahlgrenska Science Park, Medicinaregatan 8A, 413 46 Gothenburg, Sweden; 3 Centre for Analysis and Synthesis, Department of Chemistry, Lund University, POB 124, SE-22100 Lund, Sweden; 4 Section MIG, Department of Laboratory Medicine Lund University, BMC-C1228b, Klinikgatan 28, 221 84 Lund, Sweden; 5 Present Address: King’s College London, Faculty of Life Sciences & Medicine, Department of Women & Children’s Health, Guy’s Hospital, London SE1 9RT, London, United Kingdom

## Abstract

Invasive extravillous cytotrophoblast of the human placenta expresses galectins-1, -3, and -8 *in vivo* and *in vitro*. This study aimed to investigate the potential role of galectin-3 in cell migration and invasion, using recombinant human galectin-3 (rhgalectin-3), small molecule galectin inhibitor I_47_, and galectin-3 silencing. HTR-8/SVneo cell migration was stimulated by rhgalectin-3 and reduced by I_47_, which could be neutralised by rhgalectin-3. Inhibitor specificity and selectivity for the galectins expressed in extravillous trophoblast were validated in solid phase assays using recombinant galectin-1, -3, -8, confirming selectivity for galectin-3. HTR-8/SVneo cell migration and invasion, and invasion by isolated trophoblast cells in primary culture were significantly reduced in the presence of I_47,_ which could be restored by rhgalectin-3. Upon HTR-8/SVneo cell treatment with galectin-3 siRNA both *LGALS3* and galectin-3 protein were dramatically decreased. Silencing of galectin-3 induced significant reduction in cell migration and invasion, which was restored by rhgalectin-3. The influence on known mediators of cell invasion, MMP2 and -9, and integrins α_1_, α_5_, and β_1_ was followed in silenced cells, showing lower levels of MMPs and a large reduction in integrin subunit β_1_. These results show that galectin-3 acts as a pro-invasive autocrine/paracrine factor in trophoblast *in vitro*.

## Introduction

Successful pregnancy is the result of complex interactions of genetically distinct cell types regulated by various factors of maternal and fetal origin. One of the critical steps is proper invasion of the maternal decidua, myometrium and transformation of spiral arteries by extravillous trophoblast (EVT) of fetal origin^1^. Various molecules have been shown to influence trophoblast cell migration and invasion. Proteases, including matrix metalloproteinase (MMP) 2 and -9, were found to be particularly relevant for trophoblast invasion^2,3^. It is well documented that transmembrane integrin receptors are also a major family of factors regulating cell migration^4^. The specific integrin expression pattern is critical for a normal course of differentiation of invasive trophoblast^5^. Through interaction with extracellular matrix (ECM) components, integrins generate signals for trophoblast cell migration and invasion^3,5,6^. Recent results regarding the N-glycan profile of cell membrane glycoconjugates in different trophoblast cell types through gestation suggested the possibility that interaction at the maternofetal interface could be influenced by differential glycosylation in the human trophoblast^7,8^. For example, a general feature of glycans present on β_1_ integrin heterodimers is a high polylactosamine content, specifically revealed for integrins α_5_β_1_ and α_1_β_1_^9^. The level and type of β_1_ glycosylation seems to be receptor specific, and, in the case of fibronectin receptor α_5_β_1,_ parallels reduction of invasiveness during gestation^9^.

Along the pathway of invasive trophoblast differentiation, several members of the galectin family, galectin-1, -3 and -8, are expressed by extravillous trophoblast of the cell columns of the anchoring villi in first trimester placenta^10–13^. Galectins are defined by recognition of β-galactoside moieties and their carbohydrate-recognition domain (CRD)^14^. Among the 11 galectins found in humans, galectin-3 is a structurally unique mammalian beta-galactoside-binding protein, classified as a chimera type galectin with a single CRD and a non-lectin N-terminal domain that promotes oligomerisation^15^. Galectin-3 is widely distributed in different cell types, and can be localised in the cell nucleus, cytoplasm, on the cell surface, or in the extracellular environment. Through interactions with intracellular and extracellular components galectin-3 has been implicated in various functions. Intracellular galectin-3 is involved in regulation of proliferation, differentiation, survival and apoptotic events, while extracellular galectin-3 affects numerous biological processes including cell adhesion and tumor invasion, immune cell activation, and angiogenesis^16,17^.

Together with galectin-1, the presence of galectin-3 in the human and mouse female reproductive tract has been well documented^10,18,19^. In uterine epithelia of pregnant mice, galectin-3 was found immediately after implantation, but not during the pre-implantation stage of pregnancy or in non-pregnant animals^20^. In humans, expression of galectin-3 has been described in endometrium during the window of implantation and pregnancy^21^, and in placenta^10,21,22^. Among trophoblast cells, galectin-3 was localised in villous cytotrophoblast, and the cytotrophoblast of middle and distal cell columns of anchoring villi^10^. Expression of this galectin was also reported in freshly isolated trophoblast cells, extravillous HTR-8/SVneo cells, BeWo, JAr and JEG-3 choriocarcinoma cells^10,11,23^. It has been reported that galectin-3 can be associated with some pathological conditions involving the trophoblast, such as preeclampsia, HELLP syndrome and trophoblast malignancies^24,25^.

While the relevance of galectin-1 for trophoblast invasion has been documented^11^, no data are available regarding the possible involvement of galectin-3 in human trophoblast invasion. The present study was designed to address this possibility using isolated cytotrophoblast of first trimester placentas and the HTR-8/SVneo cell line as models of invasive extravillous trophoblast, and galectin-3 inhibition by a specific small molecule I_47_, or galectin-3 siRNA silencing. We provide data from functional studies *in vitro* supportive of galectin-3 involvement in the processes of cell migration and invasion, significant for human embryo implantation.

## Results

### Galectin-3 detection, localisation and (sub)cellular distribution in HTR-8/SVneo cells

**Figure 1:**
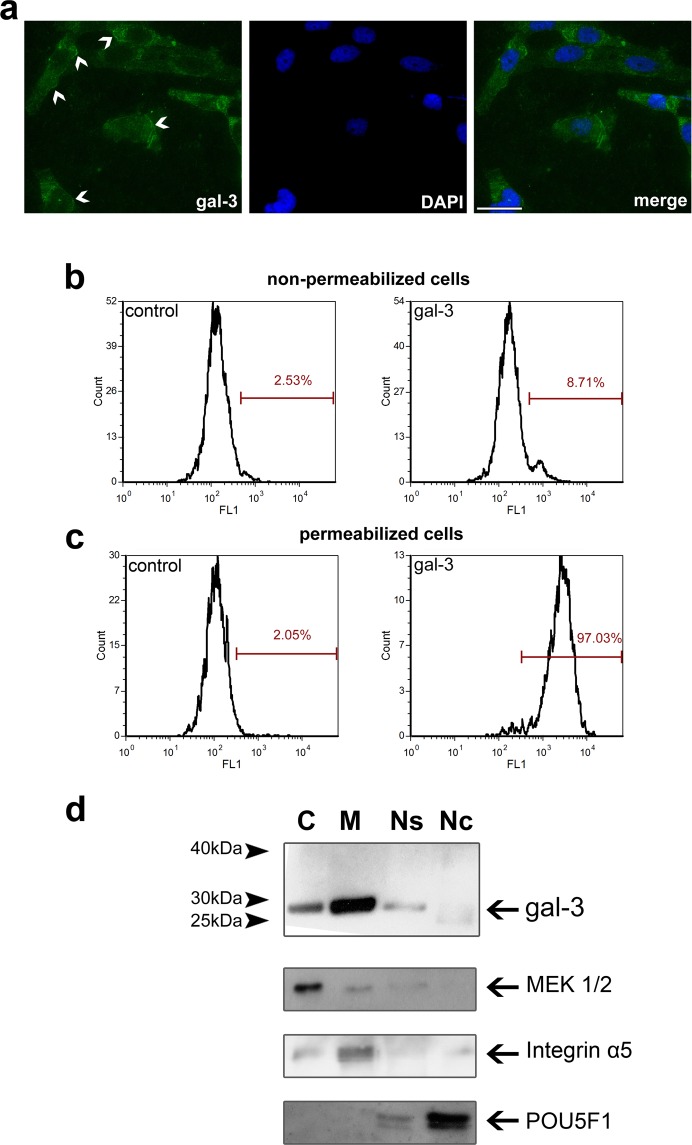
Localisation and subcellular distribution of galectin-3 in HTR-8/SVneo cells (abbreviated gal-3 in the figure). (**a**) Galectin-3 is expressed associated with the cell membrane (arrowheads) and intracellularly. Nuclei were stained with DAPI (blue); scale bar 20 µm. Non-permeabilised (**b**) or permeabilised (**c**) HTR-8/SVneo cells were probed for galectin-3 expression. The percentage of non-permeabilised or permeabilised galectin-3 positive cells is shown in each histogram; control – isotype-matched control IgG. (**d**) Galectin-3 in HTR-8/SVneo cellular compartments. Subcellular fraction purity was demonstrated using antibodies against marker proteins MEK1, α_5_ integrin, and POU5F1. The abbreviations for subcellular fractions are: C – cytoplasmic, M – membrane, Ns – nuclear soluble, Nc – nuclear chromatin. Molecular masses are indicated in kDa.

Expression of galectin-3 has been previously documented for villous cytotrophoblast, cell columns, isolated cytotrophoblast and trophoblast derived cell lines^10,11,23^. Here, the expression pattern and subcellular distribution of galectin-3 in HTR-8/SVneo cells was further examined using polyclonal anti-galectin-3 antibodies. Galectin-3 was present at the plasma membrane and in cytoplasm, as evidenced by fluorescence cytochemistry in Fig. 1a. Flow cytometric analysis showed that ~9% of non-permeabilized (Fig. 1b) and ~97% of permeabilised (Fig. 1c) HTR-8/SVneo cells were galectin-3 positive. Subcellular distribution of galectin-3 was investigated by immunoblot analysis of the fractions obtained (Fig. 1d). Galectin-3 appeared as a band of ~30 kDa in membrane, cytoplasmic, nuclear soluble and nuclear chromatin fractions (Fig. 1d), which is in line with the previously recorded presence of galectin-3 in the nucleus, cytoplasm and at the cell surface of other cell types^16^. Data from the Western blot (WB) regarding relative galectin-3 content showed that 64% of this lectin was found in the membrane fraction (comprised of solubilised plasma membrane and intracellular membranes), 19.5% in the cytoplasm, 12% in the nuclear soluble and 4.5% in the nuclear chromatin fraction. Purity of the subcellular fractions was demontrated using antibodies against marker proteins MEK1/2, α_5_ integrin and POU5F1 (Fig. 1d).

### Selective inhibition of galectin binding

We investigated the possibility that galectin-3 participates in processes relevant for trophoblast function *in vitro* using two approaches: (1) by inhibition of galectin-3 lectin function with I_47_, a thiogalactoside inhibitor of galectin-3 carbohydrate binding site and (2) by transient galectin-3 knockdown using siRNA.

**Figure 2:**
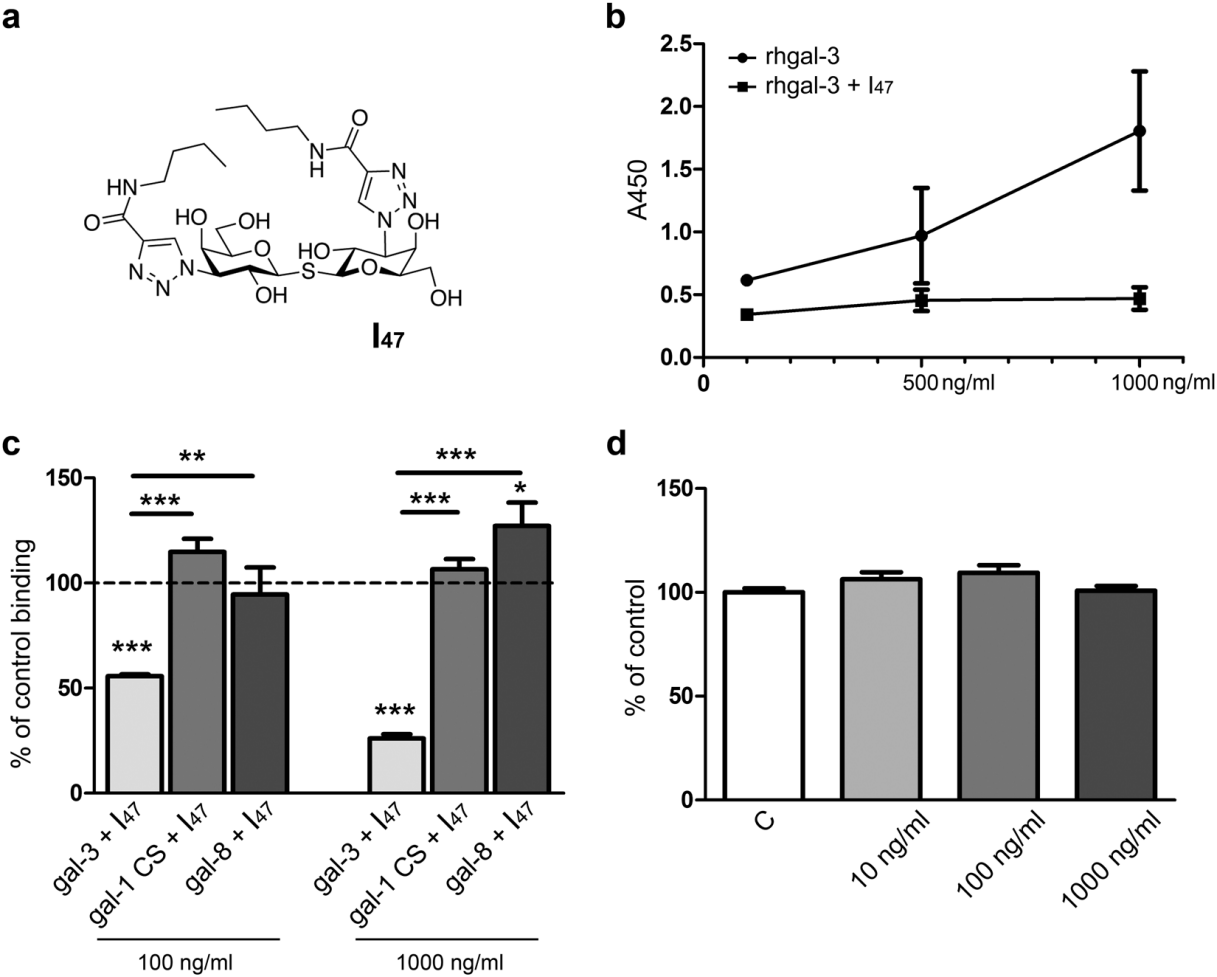
Effect of inhibitor 47 (I_47_) on binding of rhgalectin-3, CS-galectin-1 and rhgalectin-8 to Matrigel glycoconjugates in solid phase assay (abbreviated gal-1, -3, -8 in the figure). Inhibitor 47 (**a**) at 1,000 ng/ml reduces binding of rhgalectin-3 (100, 500 and 1,000 ng/ml) to immobilised glycoconjugates (**b**). Compared to rhgalectin-3 binding (at 100 and 1,000 ng/ml, both reduced from control), interaction of CS-galectin-1 (100 and 1,000 ng/ml) or rhgalectin-8 (100 and 1,000 ng/ml) with glycoconjugates was not decreased by I_47_ (1,000 ng/ml), which was significant as shown by horizontal lines (**c**). Each determination is an average of three experiments in duplicate. (**d**) The effect of I_47_ on HTR-8/SVneo cell viability. Cells were maintained for 24 h without or with different concentrations of I_47_ (10, 100 and 1,000 ng/ml). Data from two independent experiments with six replicates are expressed as percentages of untreated control values ± SEM. Data passed Kolmogorov-Smirnov normality test and were analysed by one-way analysis of variance (ANOVA) with Tukey post-hoc test (α = 0.05). Differences were significant at *p < 0.05, **p < 0.01, and at ***p < 0.001.

The selectivity of I_47_ and its effect on HTR-8/SVneo cell viability were tested in preliminary experiments. At 1,000 ng/ml, I_47_ (Fig. 2a) was found to significantly reduce binding of rhgalectin-3 to immobilised Matrigel glycoconjugates in solid phase assay (Fig. 2b) at the tested concentrations of rhgalectin-3 (100, 500, and 1,000 ng/ml). The I_47_, present in large excess and with high affinity for galectin-3, was able to prevent further binding of rhgalectin-3 at increasing concentrations to a complex mixture of ECM components contained in Matrigel coating. Little change from the baseline absorbance (A_450_ 0.2) with 0 ng/ml of rhgalectin-3 was detected with higher concentrations. Previously, some of the galectin-3 inhibitors were found to also bind one or more of the members of the galectin family, thus binding to other galectins expressed by the invasive trophoblast was tested here. To that end galectin-1, in form known as CS-galectin-1 mutant form, previously documented to maintain lectin acitivity, sugar binding specificity and affinity^26^, and rhgalectin-8 were tested for binding with or without the inhibitor I_47_. Binding to Matrigel glycoconjugates, incubated at the galectin concentrations of 100 and 1,000 ng/ml was not reduced in the presence of I_47_ (1,000 ng/ml; Fig. 2c), and in case of galectin-8, a currently poorly understood increase in binding of galectin-8 at 1,000 ng/ml only was observed. This inhibitor had no effect on HTR-8/SVneo cell viability (Fig. 2d), when the MTT test was performed with I_47_ concentrations of 10, 100 and 1,000 ng/ml. Taken together, these results demonstrate that I_47_ is a selective galectin-3 inhibitor, with no effect on HTR-8/SVneo cell viability, which makes it suitable at all studied concentrations for the functional tests shown below.

### Trophoblast cell migration is influenced by availability of galectin-3

**Figure 3:**
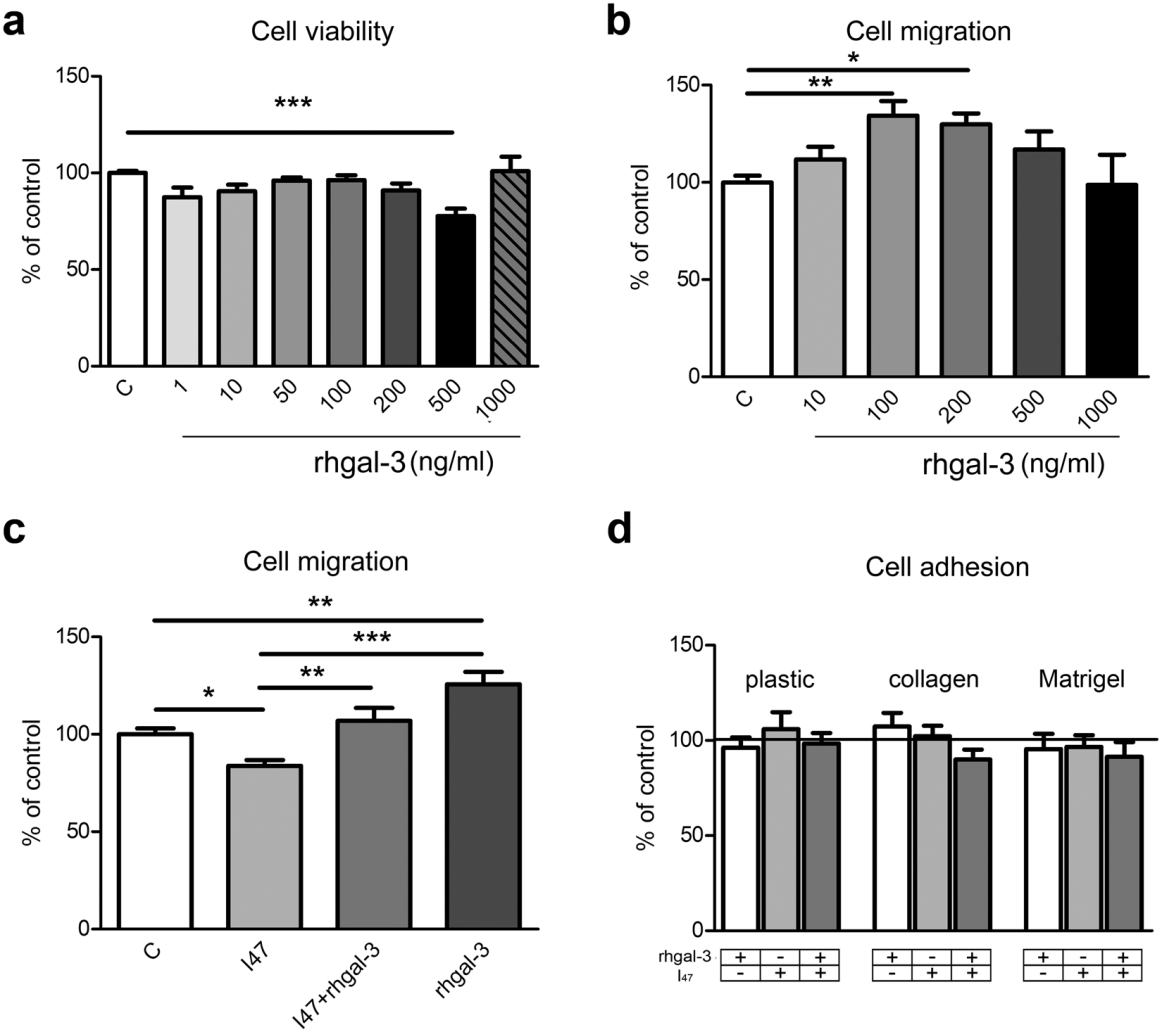
The effect of exogenously added rhgalectin-3 on HTR-8/SVneo cell viability, adhesion and migration (abbreviated gal-3 in the figure). (**a**) In the cell viability assay, HTR-8/SVneo cells were maintained for 24 h without or with different concentrations of rhgalectin-3 (1, 10, 50, 100, 200, 500 and 1,000 ng/ml). Values are given as percentages of control values (mean ± SEM), two experiments with six replicates each. (**b**) Concentration dependence of rhgalectin-3 effect on HTR-8/SVneo extravillous trophoblast cell migration. In wound healing experiments the distances crossed after 24 h period were measured using an electronic grid; data from three experiments in duplicate are presented as percentages of untreated controls, given as mean ± SEM. (**c**) HTR-8/SVneo cell migration in response to I_47_ (1,000 ng/ml) and rhgalectin-3 (200 ng/ml) alone, or in combination (200 ng/ml rhgalectin-3 + 1,000 ng/ml I_47_). Data from three experiments in triplicate are presented as percentages of untreated controls, given as mean ± SEM. (**d**) Adhesion was studied after 1 h using rhgalectin-3 (200 ng/ml), I_47_ (1,000 ng/ml) alone, or in combination (200 ng/ml rhgalectin-3 + 1,000 ng/ml I_47_), using plastic, or collagen type I, and Matrigel coated surfaces, as indicated. Data from three experiments in triplicate are expressed as percentages of the untreated control. The data were analyzed using the one-way analysis of variance (ANOVA) with Tukey or Newman-Keuls post hoc-test (α = 0.05) after passing Kolmogorov-Smirnov normality test. Differences were significant at *p < 0.05, **p < 0.01, and at ***p < 0.001.

Several lines of evidence have pointed to the relevance of galectin-3 in cell adhesion and motility^27,28^. This possibility was tested in HTR-8/SVneo cells using cell adhesion and migration assays (Fig. 3). The involvement of galectin-3 in trophoblast cell migration was evaluated by wound healing assay. When HTR-8/SVneo cell migration was determined in conditions of increased availability of rhgalectin-3 (10, 100, 200, 500 and 1,000 ng/ml), a bell-shaped curve was observed (Fig. 3b). Significant stimulatory action of galectin-3 on cell migration was obtained at 100 and 200 ng/ml, to 134% and 130% of control values respectively. To rule out a possibility that the observed effect resulted from increased cell number/viability after treatment with rhgalectin-3, a MTT assay was performed in parallel. Exogenously added rhgalectin-3 decreased viable cell number significantly at 500 ng/ml to 77% of the control value (Fig. 3a). Thus, the cell migration increase with galectin-3 was not a result of increased cell numbers or metabolic activity. In addition, the cell migration test used is based on measuring the distance crossed by viable cells during the studied interval, and the obtained result suggests that individual cells were stimulated by galectin-3 to migrate further than in control. In order to assess lectin-type interactions in HTR-8/SVneo cell migration, cells were incubated with I_47_ alone, in combination with rhgalectin-3 (Fig. 3c), or upon galectin-3 silencing (Fig. 5a). As opposed to the effect of rhgalectin-3, the presence of I_47_ (1,000 ng/ml) significantly decreased HTR-8/SVneo cell migration to 80% of the control level. When rhgalectin-3 (200 ng/ml) was added together with I_47_, inhibition of cell migration was abolished (Fig. 3c). Taking into consideration the concentrations of both I_47_, present in large excess, and rhgalectin-3 added at nanomolar concentration, it can be proposed that added galectin-3 was able to restore migration by acting jointly with HTR-8/SVneo cell released galectin-3, preferentially binding cell membrane ligands that contribute to cell migration. The inhibitor, however, may have a more restricted access to cell membrane/ECM binding sites in other cell based methods. This finding also shows that galectin-3 contributes to cell migration of HTR-8/SVneo cells through lectin-type interactions, being inhibitable by the inhibitor of the carbohydrate recognition domain of galectin-3, I_47_. In galectin-3 silenced HTR-8/SVneo cells cell migration was also reduced to 74% (Fig. 5a), which was restored by adding rhgalectin-3 (200 ng/ml) to 91% of control. Adhesion of HTR-8/SVneo cells was studied using plastic or ECM coated surfaces (Fig. 3d). HTR-8/SVneo cells were pretreated and incubated for 1 h in the absence (control) or presence of rhgalectin-3 (200 ng/ml) and I_47_ (1,000 ng/ml) separately or combined. Regardless of the treatment or surface no difference in cell adhesion was observed.

### Endogenous and added recombinant galectin-3 affect trophoblast cell invasion

**Figure 4:**
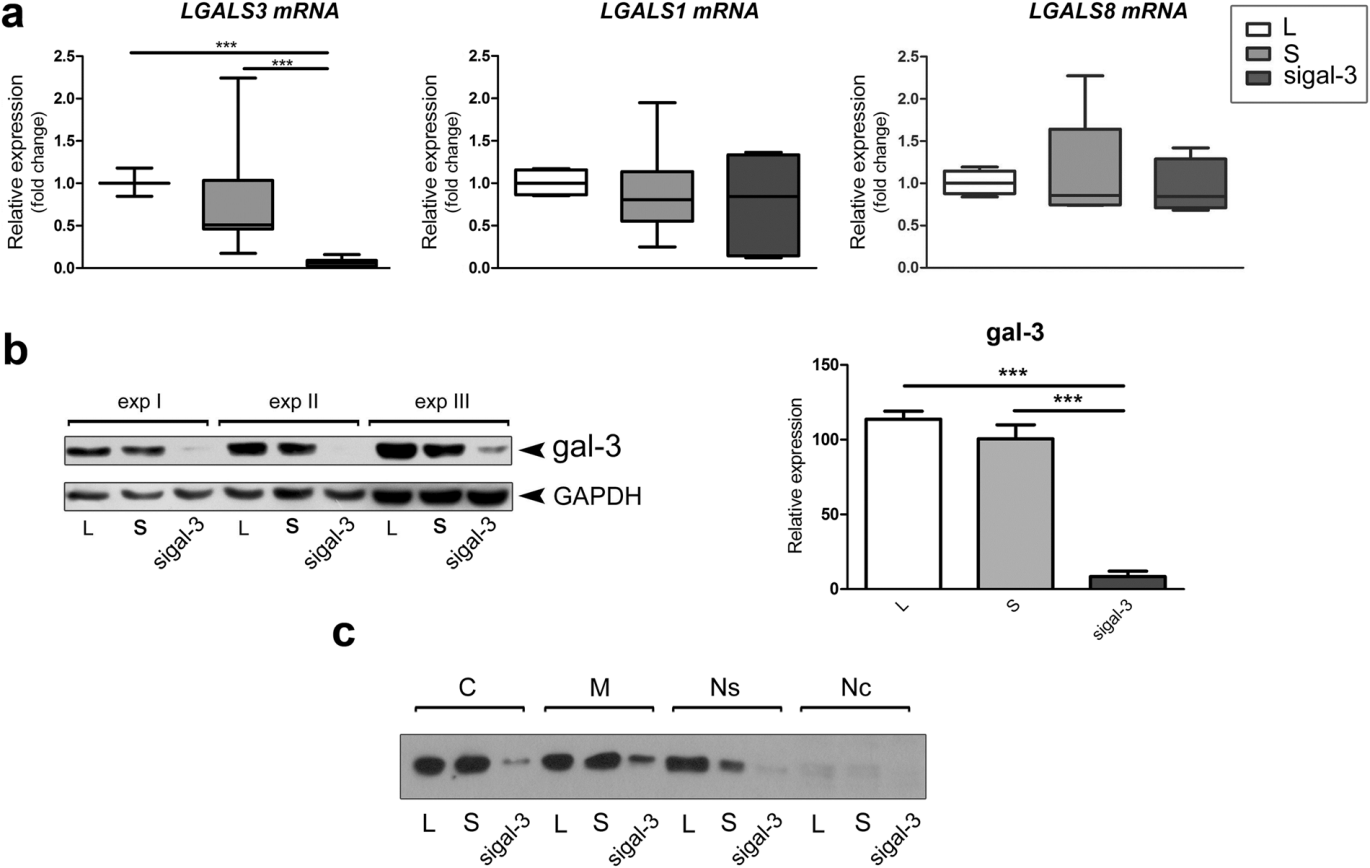
Down-regulation of galectin-3 by siRNA in HTR-8/SVneo cells (abbreviated gal-3 in the figure). Silencing efficiency was analysed by qPCR (**a**) and Western blot (**b**) in control (L, open bars), scramble-transfected (S, light gray bars) and galectin-3 transfected (sigal-3, dark grey bars). *LGALS3* was significantly reduced in galectin-3 silenced cells, while it had no effect on *LGALS1* (**a** middle) or *LGALS8* expression (**a** right), as evidenced by qPCR. One-way analysis of variance (ANOVA) with Tukey post-hoc test (α = 0.05) was used when data passed Shapiro-Wilk normality test (*LGALS1* and *LGALS8*). When data didn’t pass normality test (*LGALS3*), non-parametric Kruskal-Wallis test was used followed by Dunn’s Multiple Range test (α = 0.05) for post-hoc comparisons. (**b**) Western blot analysis of galectin-3 in control, scramble-transfected and galectin-3 transfected cells, using glyceraldehyde 3-phosphate dehydrogenase (GAPDH) as the loading control, showed significant reduction of galectin-3 compared to the control (S). (**b**) The graph gives the average of three independent experiments. Data passed Kolmogorov-Smirnov normality test and were analysed by one-way analysis of variance (ANOVA) with Tukey post-hoc test (α = 0.05). (**c**) Galectin-3 in cellular compartments in galectin-3 transfected HTR-8/SVneo cells. The abbreviations for subcellular fractions are: C – cytoplasmic, M – membrane, Ns – nuclear soluble, Nc – nuclear chromatin. Differences were significant at ***p < 0.001.

The relevance of galectin-3 for trophoblast cell invasion was studied using the Matrigel invasion assay by isolated cytotrophoblast, which most faithfully replicates extravillous trophoblast *in vivo*, and HTR-8/SVneo cells, either treated with I_47_ or specific galectin-3 siRNA. After transfection with siRNA targeting galectin-3, silencing was confirmed by qPCR and WB (Fig. 4). The expression levels of *LGAL3* and galectin-3 protein were significantly decreased to 6% and 7.6%, respectively, of control levels (Fig. 4a,b), showing high efficiency of galectin-3 silencing. Expression levels of *LGAL1* and *LGAL8* were not affected by transfection targeting galectin-3 (Fig. 4a), demonstrating the selectivity of silencing.

**Figure 5:**
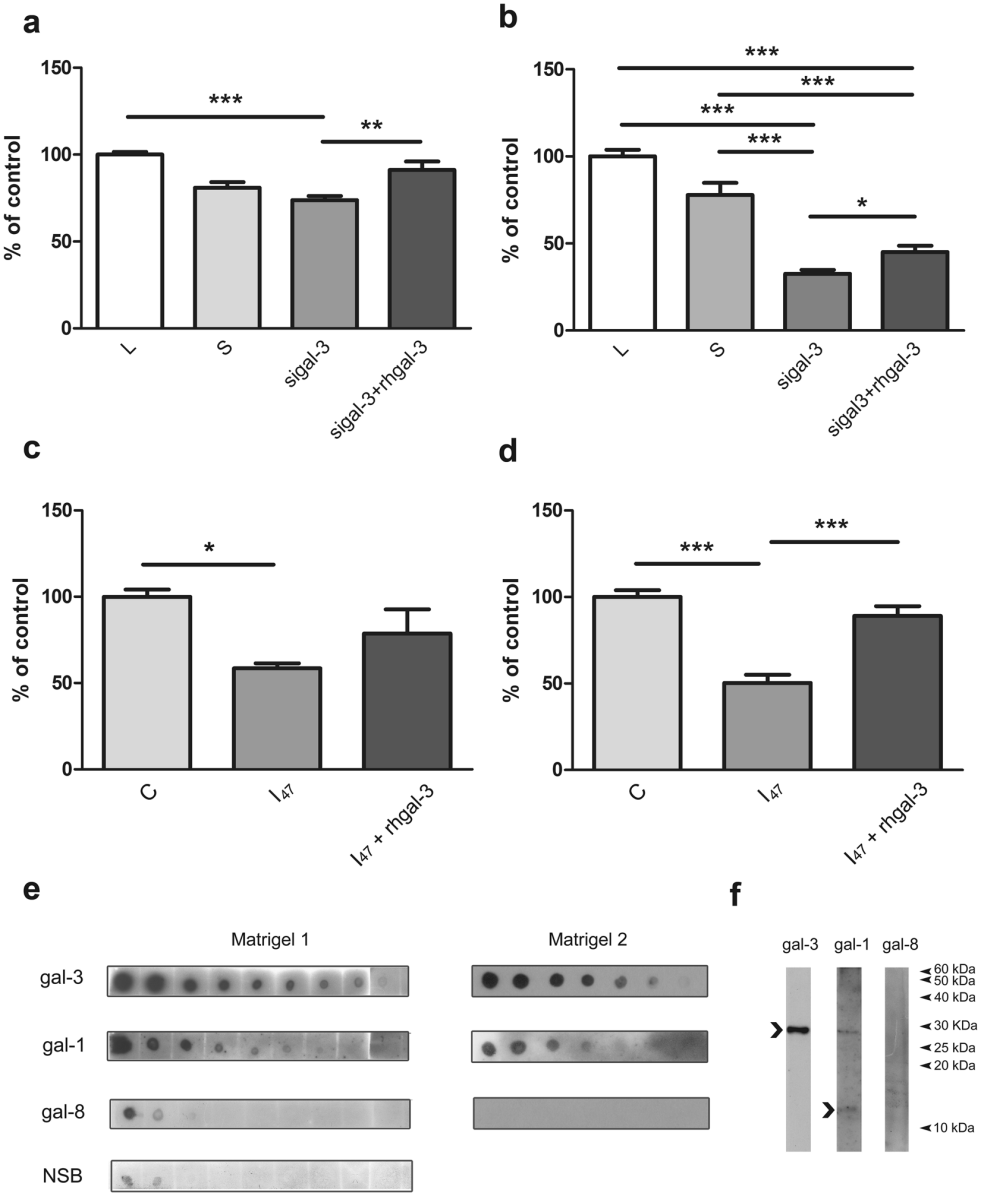
Influence of galectin-3 on trophoblast cell migration and invasion (abbreviated gal-3 in the figure). (**a,b**) Migration and invasion assays were performed with transfected cells, including lipofectamine (L), scrambled siRNA (S), galectin-3 transfected group alone (sigal-3) or in combination with exogenously added rhgalectin-3 (sigal-3 + rhgal-3). Cell migration (**a**) and invasion (**b**) by sigalectin-3 cells were reduced compared to the controls. Addition of rhgalectin-3 to silenced cells significantly reduced inhibition of cell migration and invasion. The effect of I_47_ on HTR-8/SVneo (**c**) and isolated CT (**d**) in Matrigel cell invasion test. Trophoblast cells were incubated with 1,000 ng/ml of I_47_ alone or in combination with rhgalectin-3 (200 ng/ml rhgalectin-3 + 1,000 ng/ml I_47_), showing inhibition with I_47_, which was abolished with addition of rhgalectin-3. (**b**–**d**) Cells on the underside of the filters and occupied pores were counted after 24 h culture and the data in the treatment are expressed as a percentage of the control value, given as mean ± SEM. The experiments were performed three times in duplicate (**b**–**d**). For CT invasion assay cells isolated from three individual placenats were used in three separate experiments, treatment groups with 2 replicates. (**a–d**) Data passed Kolmogorov-Smirnov normality test and were analysed by one-way analysis of variance (ANOVA) with Tukey post-hoc test (α = 0.05). Commercial Matrigel used in invasion assays was analyzed for galectin-3, -1 and -8 by dot-blot (**e**) and by Western blot (**f**). Molecular masses are indicated in kDa. Differences were significant at *p < 0.05, **p < 0.01, and at ***p < 0.001.

In the Matrigel invasion assay, trophoblast HTR-8/SVneo cells with down-regulated galectin-3 expression exhibited significantly reduced invasive ability down to 33% of the control level (Fig. 5b). When galectin-3 (200 ng/ml) was added to the system, inhibition of invasion with silenced galectin-3 cells was reduced to 45% of the control. The presence of I_47_ decreased invasion by HTR-8/SVneo cells to 60% and cytotrophoblast (CT) to 50% of the control value (Fig. 5c,d, respectively). The addition of rhgalectin-3 (200 ng/ml), in a model system where both cell released and ECM contained galectin-3 are present, resulted in reversion of the effect of I_47_, to 79% in HTR-8/SVneo (Fig. 5c) and 89% CT cells (Fig. 5d). Taken together, the data demonstrate involvement of galectin-3 in trophoblast cell invasion.

In all invasion assays performed Matrigel, the basement membrane product composed of laminin, fibronectin and proteoglycans, was used^29^. In contrast to previously published data^30^, dot blot and WB analysis detected galectin-3 and galectin-1 in all tested basement membrane preparations, as bands of ~30 kDa and 14.5 kDa, respectively (Fig. 5e,f). In addition, the 30 kDa band consistent with galectin-1 dimer was also detected. Galectin-8 was only barely visible in one of the three tested Matrigel preparations.

### Galectin-3 targeted siRNA treatment affects β_1_ integrin and MMP2 and MMP9 levels in extravillous trophoblast cells

**Figure 6:**
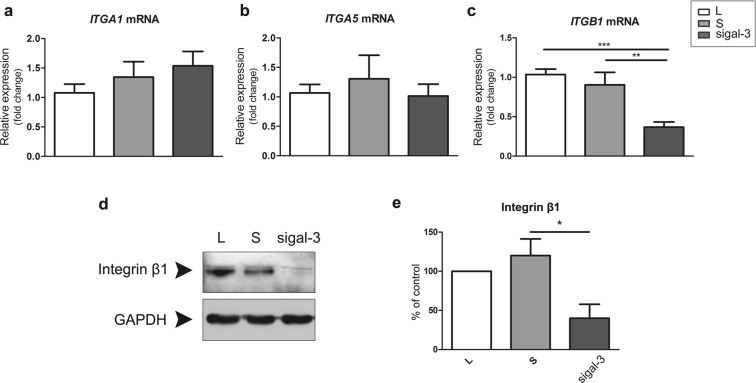
Galectin-3 (abbreviated gal-3 in the figure) silencing reduces *ITGB1* and integrin β_1_ protein level. As evidenced by qPCR, galectin-3 downregulation significantly reduced *ITGB1* (**c**), with no detected changes of *ITGA1* and *ITGA5* (**a**,**b**). Data from ten experiments passed Shapiro-Wilk normality test and were analysed by one-way analysis of variance (ANOVA) with Tukey post-hoc (α = 0.05). (**d**) Expression of integrin β_1_ in HTR-8/SVneo cells after transfection was investigated by Western blot, showing a substantial decrease of this integrin subunit. A representative experiment is shown; GAPDH served as the loading control. (**e**) The graph shows the average of three independent experiments; differences versus control (S). Data passed Kolmogorov-Smirnov normality test and were analysed by one-way analysis of variance (ANOVA) with Tukey post-hoc test (α = 0.05). In all graphs open bars represent control (L), light gray bars scramble-transfected (S) and dark grey bars galectin-3 transfected (sigal-3). Differences were significant at *p < 0.05, **p < 0.01, and at ***p < 0.001.

To explore the underlying mechanism diminishing trophoblast invasion by galectin-3 down-regulation, molecules relevant for trophoblast cell invasion were investigated. These included integrin subunits α_1_, α_5_ and β_1_ and MMP2 and −9. In the transfected cells, *ITGB1* was significantly decreased to 38% of the control value (Fig. 6c), while *ITGA1* and *ITGA5* gene expressions were unaltered (Fig. 6a,b). Protein level of the β_1_ subunit was also reduced to 40% in tranfected cells, in comparison to the control value, as evidenced by WB (Fig. 6d,e).

**Figure 7:**
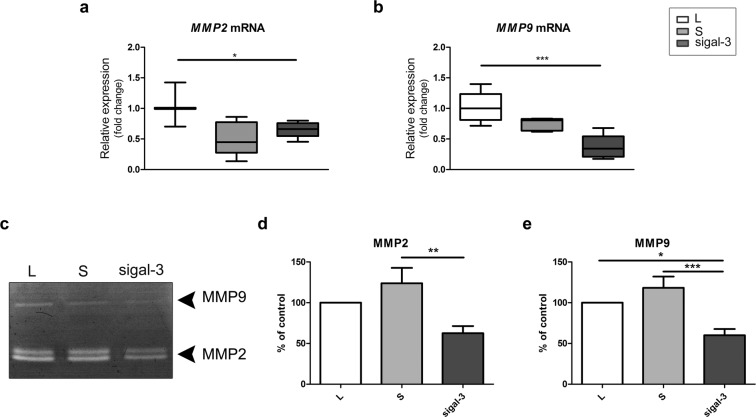
Galectin-3 silencing changes levels of MMP2 and MMP9 in HTR-8/SVneo cells (abbreviated gal-3 in the figure). The effect was studied at mRNA level for both gelatinases, (**a**,**b**) difference significant for *MMP2* and *MMP9*, in silenced vs. lipofectamine only. The graph shows the average of ten experiments. The data didn’t pass Shapiro-Wilk normality test and non-parametric Kruskal-Wallis test was used followed by Dunn’s Multiple Range test (α = 0.05) for post-hoc comparisons. (**c**) A representative gelatin zymography after galectin-3 silencing; control (L), scramble-transfected (S) and galectin-3 transfected (sigal-3). Graphs give the average of eight independent experiments for MMP2 (**d**) and MMP9 (**e**); Open bars represent control (L), light gray bars scramble-transfected (S) and dark grey bars galectin-3 transfected (sigal-3). Data passed Kolmogorov-Smirnov normality test and were analysed by one-way analysis of variance (ANOVA) with Tukey post-hoc test (α = 0.05). Differences were significant at *p < 0.05, **p < 0.01, and at ***p < 0.001.

The effect of galectin-3 down-regulation on matrix metalloproteinases 2 and -9, was studied in HTR-8/SVneo cells by qPCR and SDS-PAGE gelatin zymography (Fig. 7a–c). Silencing of galectin-3 decreased *MMP9* gene expression to 34%, and *MMP2* level to 66% (Fig. 7a,b) of the lipofectamine control. As manifested by gelatin zymography, transfection significantly lowered levels of MMP2 to 62.5% (Fig. 7d) and MMP9 to 60% (Fig. 7e) of the respective control values (zymograms analysed shown in Supplemetary Fig. S7). In culture of sigalectin-3 HTR-8/SVneo cells, rescue for reduced extracellular galectin-3 by addition of rhgalectin-3 did not induce change in MMP2 and MMP9, or integrin β_1_ (Supplementary information, Fig. S6).

## Discussion

The data presented here for the first time document the relevance of galectin-3 for invasive trophoblast cell function *in vitro*. Galectin-3 is present in human placenta, as well as in choriocarcinoma cell lines, isolated trophoblast and the trophoblast derived HTR-8/SVneo cell line^10,11,22^. Under the experimental conditions employed here the reported dominant intracellular localisation of galectin-3 was confirmed by immunocytochemistry and flow cytometry, together with membrane staining. This finding was extended further with respect to intracellular distribution of galectin-3 between different cell compartments, showing that galectin-3 is present in nuclear chromatin, nuclear soluble, cytoplasmic and cell membrane fractions. The membrane fraction associated galectin-3 was by far the most abundant, which seems to contradict the finding for intact unpermeabilised cells evidenced by flow cytometry. This however likely stems from the different membranes analysed by the two methods. Namely, the outer aspect only of the plasma membrane of non-permeabilised cells is accessible for antibody binding in flow cytometry, as opposed to the complex mixture of disrupted plasma, mitochondrial and ER/Golgi membranes after cell fractionation. Considering plasma membrane, the two methods used can detect galectin-3 at distinct locations, since galectin-3 engaged in lipid rafts, trapped in extracellular lattices, or within the cytoplasmic aspect of the plasma membrane documented for other cell types^31,32^ could be inaccessible for antibody binding in flow cytometry of trophoblast shown here. Our finding of galectin-3 in nuclear compartments is consistent with published data and galectin-3 function in early assembly of the splicing machinery for mRNA-processing and nuclear export^33^.

Based on their binding characteristics galectins are expected to influence multiple processes in cells exposed to or expressing them. Galectin-1 and galectin-3 are differentially expressed by endometrium during the menstrual cycle and early pregnancy suggestive of specific roles in embryo implantation^21^. Along with the finding that galectin-3 is absent from non-decidualised endometrium and is not present in the uterus of non-pregnant females^20^, it has been proposed that it has a pregnancy related function in the uterine environment. The interstitial trophoblast invasion characterized by interaction of dispersed EVT with decidual stromal cells and the matrix they produce is one of the main interests of our group. Decidual stromal cells of first trimester pregnancy are known for abundant expression of both galectin-1 and galectin-3^10,22^, so the invasive trophoblast *in vivo* is exposed to both intracellular and extracellular galectin-1 and -3. We have previously shown that trophoblast cell invasion *in vitro* is dependent on both endogenous and exogenous galectin-1^11^, while data from the literature support the relevance of galectin-1 for maintenance of pregnancy *in vivo*^34,35^. Based on galectin binding specificity and potentially shared ligands, it was of interest to determine whether galectin-3 participates in processes affecting trophoblast cell migration and cell invasion. Recently, we have shown that trophoblast HTR-8/SVneo cells release galectin-1, -3 and -8 into culture medium^36^. Thus, two approaches were taken, the first one with either increased, or blocked extracellular galectin-3, and the other based on reduction of intracellular galectin-3 by targeting its mRNA. Several previous studies employing mouse animal models used 33DFTG (formerly known as TD139), a synthetic inhibitor of lectin-type interactions of galectin-3^37–40^. In the present study, thiogalactoside inhibitor, I_47_ that mimics N-acetyllactosamine was used, based on its reported specificity and high affinity for galectin-3^41^. This was supported by our solid phase assay data with immobilised Matrigel glycoconjugates and rhgalectin-3, CS-galectin-1, and rhgalectin-8, showing that I_47_ decreases the binding of rhgalectin-3 only. Therefore, it was concluded that I_47_ met the initial criteria for use in cell-based assays with trophoblast cells expressing galectin-1, -3, -8.

In many cell types both endogenous and exogenous galectin-3 have been implicated in cell viability and survival^42,43^ inducing either positive or negative effects on cell proliferation and apoptosis. Hence, its influence on HTR-8/SVneo cells was assessed, showing a small, concentration dependent, reduction in cell numbers and/or metabolic activity in rhgalectin-3 treated cells. This was not accompanied with change in apoptosis rate (not shown). Neither treatment with inhibitor I_47_, nor galectin-3 silencing induced any alteration in MTT (not shown). Therefore, the influence of galectin-3 on trophoblast cell viability, survival and apoptosis was not studied further.

Galectin-3 is also known to modulate cell–ECM interactions in cell adhesion, so plastic, collagen type I, and Matrigel coated surfaces were used in our model. Irrespective of the surface no change was observed in adhesion of cells treated with rhgalectin-3, I_47_, or their combination after 1 h. In other cell types galectin-3 has been shown to either promote or inhibit cell adhesion^27,44^, or produce no change, as found here. These conflicting findings indicate cell type specific effects of this lectin on cellular adhesion molecules. On the other hand, cell migration in the wound healing test was increased in the presence of recombinant rhgalectin-3 in a bell shaped manner (maximal at 100 and 200 ng/ml). The decrease when inhibitor I_47_ was present could be reversed by rhgalectin-3. Similar concentration dependence and a maximally effective concentration were described for galectin-3 driven monocyte migration^28^. Invasive extravillous trophoblast *in vivo* would be exposed to extracellular galectin-3 from multiple sources, probably mostly released by decidual stromal cells or the trophoblast itself. Addressing the influence of extracellular galectin-3, our data showed that the small molecule inhibitor, I_47_, altered *in vitro* cell invasion. In the presence of inhibitor I_47_, both isolated cytotrophoblast and HTR-8/SVneo cell invasion was reduced, which seems to be reversible by the addition of rhgalectin-3. Inhibitory compounds that are based on disaccharides, which include I_47_, are considered relatively soluble, and not taken up well across membranes^17,45^ (and personal communication with Hakon Leffler), thus it can be assumed that predominantly/exclusively extracellular galectin-3 was blocked. There are also several lines of evidence pointing to the relevance of galectin-3 for cancer cell migration and invasion^46^. Exogenously added galectin-3 contributed to the migration of many tumor cells mainly through interaction with ECM glycoprotein partners, such as fibronectin, laminin, collagen type IV^47^. Moreover, galectin-3 induced migration and invasion of mammary carcinoma cells involved integrin clustering and ligand induced integrin activation^48^. Although it is possible that the same can apply to normal human trophoblast migration and invasion and that our results indicate the relevance of lectin type interactions for this process, the molecules involved in I_47_ inhibition remain to be elucidated. When galectin-3 was targeted by siRNA drastic reduction specific for this galectin was induced, resulting in decreased cell migration and invasion, which could be restored by addition of rhgalectin-3 (Fig. 5a,b). Analysis of galectin-3 in cell fractions after galectin-3 silencing revealed less galectin-3 in all compartments, leaving the possibility for reduced galectin-3 actions in these cells. In that context it is interesting to note that Matrigel, a commercial ECM widely used to study cell migration and invasion in a wide range of models, harbours certain galectins, and galectin-3 in particular (Fig. 5e,f), thus participating in base line cell invasion. The Matrigel galectin-3 contribution to trophoblast cell invasion shown here is however hard to fully appreciate, since HTR-8/SVneo cells also release galectin-3 into the medium^36^. On the other hand, the presence of galectins in Matrigel could be seen as a factor making this matrix a better model of decidual ECM. Nevertheless, the presence of galectin-3 specific inhibitor significantly reduced invasion of Matrigel by isolated cytotrophoblast and HTR-8/SVneo cells (Fig. 5c,d). The interaction of galectins with the ECM is however of great interest, since galectin-1 has been shown to influence T-cell survival differently depending on its presentation, whether soluble, or cell membrane and matrix bound^30^.

Based on the results of functional tests, it was further undertaken to determine whether any of the molecules known to participate in trophoblast cell migration/invasion, such as integrins and gelatinases (MMP2 and -9), were involved in reduction observed in galectin-3 silenced cells. Among the tested integrin subunits α_1_, α_5_, β_1_, forming receptors for fibronectin, collagen type IV and laminin, subunit β_1_ was the only one that was significantly reduced at the RNA and protein levels with galectin-3 silencing. This finding is of particular interest since subunit β_1_ is the binding partner of a large number of α subunits, thus forming ECM receptors that are expressed as trophoblast cells acquire physiological invasiveness *in vivo*. Less integrin subunit β_1_ could thus lead to a decrease in cell migration/invasion. Our finding that silencing endogenous galectin-3 causes a decline in levels of integrin β_1_ suggests considerable relevance of their interaction for the trophoblast. This concurs with the reports that galectin-3 promotes healing during re-epithelialization of corneal wounds and cell migration of a corneal HCLE cell line, by involving the β_1_ integrin dimer α_3_β_1_ as a binding partner^49,50^. Other factors modulating trophoblast invasion have also been associated with changes in β_1_ integrins, such as triiodothyronine^51,52^. Previously, galectin-3 silencing was associated with another integrin subunit, β_3_, in an endometrial cell line RL95-2^53^. In cancer biology galectin-3 is overexpressed in migrating cells during wound healing, and during tumor invasion and metastasis^49,54^. Mechanisms of action by which galectin-3 can affect the intrinsic motility of cells include binding or upregulation of integrins, as a group of cell surface proteins that mediate cell adhesion to the extracellular matrix^50,55^.

Cellular invasion requires proteolytic degradation of extracellular matrix molecules. The MMP system is a well-documented effector mechanism important for trophoblast invasion^2,56^. Matrix metalloproteinases, MMP2 and MMP9, have been shown to mediate the influence of various cytokines and growth factors on trophoblast cell invasion^52,57,58^. MMP9 is able to degrade type IV collagen of the basement membrane, fibronectin, laminin, elastin, entactin, gelatin, and proteoglycans^59,60^. The regulation of MMPs is believed to occur at the transcriptional level, by activation of latent proenzymes, and by inhibition of proteolytic activity^58^. In this study, a decrease in *MMP2* and *MMP9* mRNA and reduced level/gelatinolytic activity of MMP2 and MMP9 in the conditioned media of galectin-3 silenced cells were observed, pointing to the significant contribution of post-transcriptional regulation of MMP expression. This could be due to direct interaction of these molecule families, since all MMPs have potential glycosylation sites. Across the MMP family, conserved glycosylation sites appear mainly as N-glycosylations in the active site, which is compatible with the potential ligand role for MMP. Thus, decrease in intracellular galectin-3 could affect potential MMP binding and export to the cell membrane^61^. In addition, both molecules that function upstream (regulators) and those that function downstream (e.g. substrates) of MMPs are subject to glycosylation^61^. Alterations in the glycosylation status of these molecules, as well as the presence of binding lectins, might have direct or indirect implications on MMP function.

Others have reported that altered MMP levels may involve changes in mRNA half-life^62^ and decreases in *MMP* stability^63^. Destabilization of mRNA is a possible post-transcriptional regulatory mechanism by which MMP2 and MMP9 activity is reduced in galectin-3 silenced trophoblast derived cells. However, the exact mechanism(s) for post-transcriptional MMP2 and MMP9 regulation by galectin-3 still remains to be elucidated. It is of interest to note that in other cell types galectin-3 has been associated with MMPs through EMMPRIN (extracellular matrix metalloproteinase inducer), also known as CD147 and basigin, a member of the immunoglobulin family that is present on the surface of tumor cells, which stimulates nearby fibroblasts to synthesize MMPs^64^. Thus, concomitant stimulation and colocalisation of galectin-3 with CD147 are associated with increased gelatinolytic activity in the actively ulcerating human cornea^65,66^. The highly glycosylated species of CD147 are responsible for the induction of matrix metalloproteinases^67^ with β1, 6-branched polylactosamine residues constituting major components in this molecule^68^. Since these glycan residues are unique in that they mediate cell motility via modulation of carbohydrate dependent interactions between galectin-3 and many cell surface and extracellular molecules, such as fibronectin, laminin, and integrins, the involvement of CD147 and other molecules in invasive trophoblast needs to be investigated further. So far, the highly glycosylated 65 kDa EMMPRIN has been reported to participate in activation of MMPs during labor at term^69^.

Data regarding the clinical relevance of galectin-3 for normal pregnancy have only started to accumulate. Thus, it was shown that galectin-3 was significantly down-regulated in the extravillous trophoblast of IUGR placentas^70^. Should galectin-3 involvement in pregnancy be further supported by clinical data in various conditions pertinent to pregnancy outcome, the possibility to neutralize it by small molecule inhibitors, as shown here, may provide a useful experimental tool. At this point, it can be concluded that galectin-3 is relevant for invasive trophoblast *in vitro*, as we previously showed for galectin-1. Given the huge potential of these galectins to interact with diverse molecules, much remains to be learned about the roles galectins may play in the placental bed.

## Materials and Methods

### Reagents and antibodies

RPMI 1640 and DMEM/F12 media, antibiotic/antimycotic solution and fetal calf serum (FCS) were obtained from Gibco (Paisley, UK). TRI Reagent, primers and dNTPs were from Applied Biosystems by Life Technologies (Carlsbad, CA). Oligo(dT) 12–18 primers were from Invitrogen (Carlsbad, CA, USA). RevertAid reverse transcriptase was obtained from Fermentas, Inc., (Vilnius, Lithuania). Lipofectamine RNAiMAX was from Invitrogen and Opti-MEM reduced medium was obtained from Gibco. Galectin-3 Silencer Select siRNA (s8148) and Silencer Negative Control siRNA #2 (AM4613) were purchased from Ambion (Thermo Fisher Scientific Inc., Fremont, CA, USA).

The inhibitor of the galectin-3 carbohydrate binding domain di-(3-deoxy-3-(4-((butylamino) carbonyl)-1H-1,2,3,-triazol-1-yl)-β-D-galactopyranosyl) sulfane inhibitor 47 (I_47_, C_26_H_42_N_6_O_10_S, Kd = 29 nM ± 7 nM) synthesized as previously described^40^ was generously provided by Galecto Biotech AB. Inhibitor I_47_ was used to block lectin activity of extracellular galectin-3 when present, irrespective of its origin (secreted by the cells, or supplemented recombinant galectin-3). The purity was determined to be >99% according to HPLC-analysis (Agilent series 1100 system, column Eclipse XDB-C18, 0.8 mL/min H2O-MeCN gradient 5–95% 13 min with 0.1% trifluoroacetic acid). Recombinant human galectin-3 (rhgalectin-3), recombinant human galectin-8 (rhgalectin-8), goat anti-galectin-3 (AF1154) and goat anti-galectin-8 (AF1305) antibodies were from R&D Systems (Abingdon UK). The mutant form of galectin-1 in which all six cysteine residues were replaced by serine (CS-galectin-1) and rabbit anti-galectin-1 were obtained from Dr Toshihiko Kadoya, Maebashi Institute of Technology (Japan). Rabbit anti-integrin β_1_ (AB1952) was from Chemicon (Temecula, CA, USA) and rabbit anti-MEK1/2 (RB-1662-P0) was from Thermo Fisher Scientific Inc., (Fremont, CA, USA). Rabbit anti-Oct-3/4 (sc-9801) and rabbit anti-integrin α_5_ (sc-10729) were obtained from Santa Cruz Biotechnology Inc., (Santa Cruz, CA, USA). Anti-goat IgG antibody AlexaFluor 488 was from Invitrogen/Molecular Probes/Life Technologies (Thermo Fisher Scientific Inc., Carlsbad, CA, USA). Biotinylated horse anti-goat IgG, avidin-biotinylated peroxidase complex (ABC), Vectashield mounting medium with DAPI were from Vector Laboratories (Burlingame, CA, USA).

All other reagents were of the best commercial grade available.

### Cell culture

The extravillous trophoblast cell line, HTR-8/SVneo, was kindly provided by Dr Charles H Graham (Queen’s University, Kingston, ON, Canada) and cultured in RPMI 1640 supplemented with 5% FCS (v/v) and antibiotic/antimycotic solution. The cell line was authenticated by the European Collection of Authenticated Cell Cultures (ECACC) using Short Tandem Repeat analysis (supplementary, SNote) as described in 2011 in ANSI Standard (ASN-0002) Authentication of Human Cell Lines and in^71^. Cytotrophoblast cells were isolated from the first trimester of pregnancy placentas from legal abortions (6–12 weeks) undertaken for non-medical reasons, at Obstetrics and Gynecology Clinic Narodni Front, Belgrade, Serbia, using sequential trypsin digestion, Percoll gradient centrifugation, and assessment, as previosuly described^11^. Briefly, after isolation, cells were identified as trophoblast by immunocytochemical staining for cytokeratin-7 (CK-7), and preparations with 95% cytokeratin positive cell were used. In addition, a small proportion of CD45-positive cells were removed using immunomagnetic beads prior to invasion assay. Cytotrophoblast was cultured in DMEM/F12 supplemented with 10% FCS and with antibiotic/antimycotic solution. This study was conducted according to local ethical standards (document GSP/05) and approved by Institutional Ethical Committee of the Institute for the Application of Nuclear Energy, INEP, University of Belgrade.

### Galectin-3 siRNA transfection

HTR-8/SVneo cells were seeded into 6-well or 24-well plates and grown in RPMI 1640 medium containing 5% FCS without the antibiotic/antimycotic mixture to ~60% of confluence. The galectin-3 treatment group was transfected with 30 nM galectin-3 siRNA (sigalectin-3) in Opti-MEM I GLUTAMAX I Reduced Serum Medium, while the negative control group (S) was transfected with 30 nM scrambled siRNA using Lipofectamine RNAiMAX, according the manufacturer’s instructions. The reagent-only group (L) contained only transfection reagent. HTR-8/SVneo cells were incubated for 48 h at 37 °C and quantitative real-time qPCR and WB analyses were performed. The experiments were repeated at least three times.

### Immunocytochemistry and flow cytometry

For immunocytochemical analysis, HTR-8/SVneo cells cultured on glass cover slips in the complete RPMI 1640 were fixed with ice-cold acetone–methanol (1:1). Anti-galectin-3 (5 μg/ml) was visualized with anti-goat IgG AlexaFluor 488. Slides were mounted with Vectashield mounting medium containing DAPI and examined using a Carl Zeiss Axio Imager microscope with an AxioCam HR Camera (Carl Zeiss, Jena, Germany).

Flow cytometry was used to analyse expression of galectin-3 in untreated HTR-8/SVneo cells and the effect of rhgalectin-3 treatment on M30 (a cytokeratin 18 fragment indicative of apoptosis) expression. HTR-8/SVneo cells from control or treatment groups were detached with cold 5 mM EDTA in PBS, and washed twice with PBS2 (PBS, 2% FCS, 0.01% sodium azide). Cells were permeabilized with the Fix&Perm Cell Permeabilization Kit (Invitrogen, Frederick, MD, USA) following the manufacturer’s procedure. Permeabilized and non-permeabilized cells were incubated with anti-galectin-3 or M30 antibody. After incubation and subsequent washing with permeabilization buffer for permeabilized cells (PB) or with PBS2 for non-permeabilized cells, cells were stained with anti-goat AlexaFluor 488 (galectin-3) and anti-mouse AlexaFluor 488 (M30). For assessment of non-specific binding, cells were incubated with non-immune goat IgG.

### Solid-phase assay

The solid-phase assay was performed in 96-well plates coated with Matrigel in PBS (5 µg/200 µL) overnight at 4 °C. Plates were blocked with 1% BSA for 1 h at 37 °C, and washed with PBS. Recombinant human CS-galectin-1, rhgalectin-3 and rhgalectin-8 at 0, 100, 500 or 1,000 ng/ml were added to the plates without or with I_47_ (1,000 ng/ml), and incubated overnight at 4 °C. After washing with PBS, 100 µl of respective antibody for each galectin, rabbit polyclonal anti-galectin-1 IgG (Kirin Brewery, Japan), goat anti-galectin-3 (R&D) or goat anti-galectin-8 (R&D) were added and incubated for 2 h at room temperature (RT), with shaking. Wells were washed with PBS and incubated with biotinylated goat anti-rabbit IgG (for galectin-1) and horse anti-goat IgG (for galectin-3 and galectin-8). After 30 min, wells were washed, incubated with ABC for another 30 min and further with substrate and chromogen. The reaction was stopped with 0.2 M H_2_SO_4_. Non-specific binding was estimated by omitting the specific antibody.

### Subcellular fractionation

Subcellular fractionation of HTR-8/SVneo cells was carried out using the Subcellular Protein Fractionation Kit for Cultured Cells (ThermoScientific, Rockford, USA), according to the manufacturer’s instructions. The procedure yields a cytosolic fraction (C), a membrane fraction (M), a nuclear soluble fraction (N) and a nuclear chromatin bound fraction (Nc). Protein concentration was then determined in each subcellular fraction using a BCA Protein Assay kit (ThermoScientific, Rockford, USA) and equal amounts of each fraction were loaded for SDS-PAGE electrophoresis. This analysis was performed on subcellular fractions obtained from cells cultured in complete RPMI 1640 medium and from cells after the transfection protocol. After transfer, membranes were incubated with goat anti-galectin-3. Relative galectin-3 content was calculated using densitometric evaluation of band intensities, since equal amounts of protein were used from each fraction. The obtained intensities were summed and that of each galectin-3 band was expressed as a percentage of total galectin-3. The following antibodies were used as controls for fraction purity: anti-MEK1, anti- integrin α5 and POU class 5 homeobox 1 (POU5F1, also known as OCT3/4) antibodies.

### Dot blot analysis

The presence of galectin-1, -3 and -8 in the Matrigel preparations used in the invasion assay was detected by dot blot analysis. Aliquots of serially diluted Matrigel (2 µl each) were spotted on nitrocellulose strips and dried at RT. Nonspecific binding sites were blocked with 1% BSA in PBS for 1 h, at RT. Individual strips were incubated with: anti-galectin-3, anti-galectin-1 and anti-galectin-8 antibodies overnight at 4 °C. Galectins were detected with Pierce ECL Western Blotting Substrate (Pierce Biotechnology, Rockford, IL, USA).

### SDS-PAGE and immunoblot

Subcellular fractions, HTR-8/SVneo cell lysates after transfection and Matrigel were analyzed by Western blot. For electrophoresis under reducing and denaturing conditions all samples were prepared by boiling for 5 min in 0.125 M Tris-HCl buffer, containing 4% SDS (w/v), 20% glycerol (v/v), 0.1% bromphenol blue and 10% 2-mercaptoethanol (v/v). Following electrophoresis on 10% polyacrylamide gel and transfer, the membranes were incubated with: anti-galectin-3, anti-galectin-1, anti-galectin-8 or anti-integrin β_1_ overnight at 4 °C, with constant shaking. Staining for GAPDH (cell lysates) was used as the loading control. Proteins were detected with Pierce ECL Western Blotting Substrate (Pierce Biotechnology, Rockford, IL, USA). The obtained signals were scanned and analyzed by the ImageMaster TotalLab v2.01 program (Amersham Biosciences, Inc., Piscataway, NJ, USA).

### Gelatin zymography

Matrix metalloproteinase gelatinolytic activity was studied semi-quantitatively using SDS-PAGE gelatin zymography. Gelatinase activities were determined in conditioned media HTR-8/SVneo cells transfected with sigalectin-3 siRNA, S and L group using 11% acrylamide gel containing 1 mg/mL gelatin under non-reducing conditions, with 25 µg of protein loaded per lane^51^. The experiments were carried out 8 times.

### Quantitative real-time PCR analysis

Total RNA was isolated from transfected HTR-8/SVneo cells (L, S and sigalectin-3 group) using TRIzol (Applied Biosystems, Carlsbad, CA, USA), as suggested by the manufacturer. First-strand cDNA was synthesized from 1 µg of total RNA, using 0.5 μg of Oligo(dT) 12–18 primers (Invitrogen, Carlsbad, CA, USA), 250 µM of each dNTP and 200U of RevertAid reverse transcriptase (Fermentas, Vilnius, Lithuania). Real-time PCR was performed using the 7500 Real-Time PCR System (Applied Biosystems, Carlsberg, USA). The reaction mixture contained 1 µl of cDNA, 5 µl 2x SYBR® Green PCR Master Mix (Applied Biosystems, Carlsberg, USA) and specific forward and reverse primers in a final concentration of 0.5 µM. Reactions were run at 95 °C for 10 min, followed by 40 cycles of 15 sec at 95 °C and 1 min at 60 °C. Melting curve analysis was performed to verify amplification specificity. Expression levels of *LGALS3*, *LGALS1*, *LGALS8*, *ITGA1*, *ITGA5*, *ITGB1*, *MMP2* and *MMP9*, were normalized to the housekeeping gene *GAPDH*. Calculations were made by the comparative ΔΔCt method^72^. The sequences of specific primers are given in supplemental material (STable 1).

### Cell viability assay (MTT)

The viability of HTR-8/SVneo cells was assessed as described previously^73^. HTR-8/SVneo cells were incubated for 24 h in treatment media containing different concentrations of rhgalectin-3 (1–1,000 ng/ml) or I_47_ (10, 100 and 1,000 ng/ml) in 200 µl culture medium. In experiments with I_47_, DMSO supplemented medium was used as the control. The experiment was repeated three times with six replicates for each treatment group.

### Cell adhesion

Adhesion assays were performed on plastic/uncoated wells or wells precoated with Collagen type I or Matrigel, as previously described^11^. HTR-8/SVneo cells were preincubated with rhgalectin-3 (200 ng/ml) or I_47_ (1,000 ng/ml). Experiments were repeated three times in triplicate per treatment group.

### Cell wounding and migration assay

The effect of various concentrations of rhgalectin-3 (10, 100, 200, 500 or 1,000 ng/ml), I_47_ (1,000 ng/ml), rhgalectin-3 with I_47_ and sigalectin-3 transfection on HTR-8/SVneo cell migration was investigated using the cell wounding assay as previously described^74^. HTR-8/SVneo cells were incubated until confluent, and scraped off using a sterile pipette tip. After rinsing, RPMI 1640 (containing 0.1% BSA) without or with rhgalectin-3 or I_47_ was added. The pre-selected fields were photographed at zero point and after 24 h. The distances crossed after 24 h period were measured using an electronic grid and the distances crossed by the cells were determined. The mean value for the controls was set to 100% and the data are expressed as percentages of the control value. The experiment was repeated three times in duplicate.

### Cell invasion assay

Transwell Matrigel invasion assays were conducted as described previously^11^. The invasion assay was run for 24 h using isolated CT and HTR-8/SVneo cells without or treated with I_47_, or rhgalectin-3 + I_47_. Invasion assay was also performed using HTR-8/SVneo cells transfected with sigalectin-3, as well as a negative control (S), and reagent-only (L). The treatments were prepared in DMEM/F12 for CT or RPMI 1640 for HTR-8/SVneo, both containing 0.1% BSA. Invaded CT cells were stained using anti-CK-7 antibody, and by Giemsa for HTR-8/SVneo cells. Cells on the underside of the filters and the occupied pores were counted in non-overlapping fields of the entire insert. Data presented are expressed as percentages of the control values. The experiment was repeated three times in duplicate.

### Statistical analysis

The data were analysed using GraphPad Prism Demo Software (GraphPad Software, Inc., La Jolla California USA) and evaluation detailed in figure legends.

## Supplementary information

Supplementary information

## Data Availability

The datasets generated and/or analysed during the current study are available from the corresponding author on reasonable request.
