# Supplementary material for: Human trophoblast requires galectin-3 for cell migration and invasion
Source: Sci Rep. 2019 Feb 14;9:2136. doi: 10.1038/s41598-018-38374-w (PMC6376043; doi:10.1038/s41598-018-38374-w)
Supplement: Supplementary file 1 — Supplementary information [file 41598_2018_38374_MOESM1_ESM.doc]

**Supplementary information**

Manuscript No: SREP-18-29286

**Human trophoblast requires galectin-3 for cell migration and invasion**

Ž. Bojić-Trbojević, M. Jovanović Krivokuća, A. Vilotić, N. Kolundžić, I. Stefanoska, F. Zetterberg, U. J. Nilsson, H. Leffler, Lj. Vićovac^1#^

**Supplementary Figures**


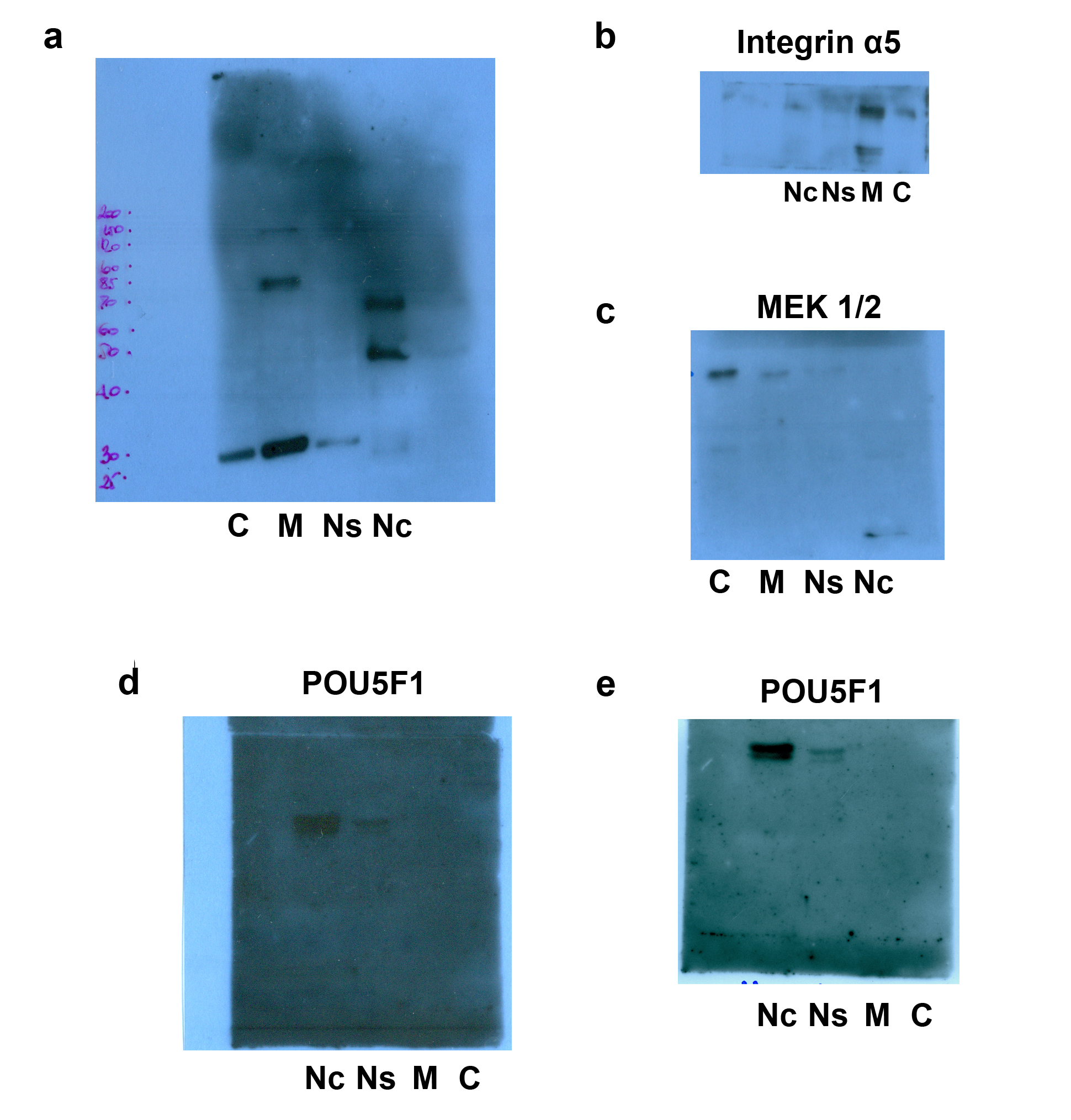


**Supplementary Figure S1**

Full-length blots of Figure 1D. a) Galectin-3 in subcellular compartments; exposition 7 minutes. Fraction purity was tested with antibodies against integrin α_5_ subunit (b), MEK1/2 (c), and POU5F1 (d and e); exposition 10 minutes. For POU5F1regular scan full-length blot (left) and the same blot after narrow field transillumination scanning (right) are represented. The abbreviations are: C-cytoplasmic, M-membrane, Ns-nuclear soluble, Nc-nuclear chromatin.

**
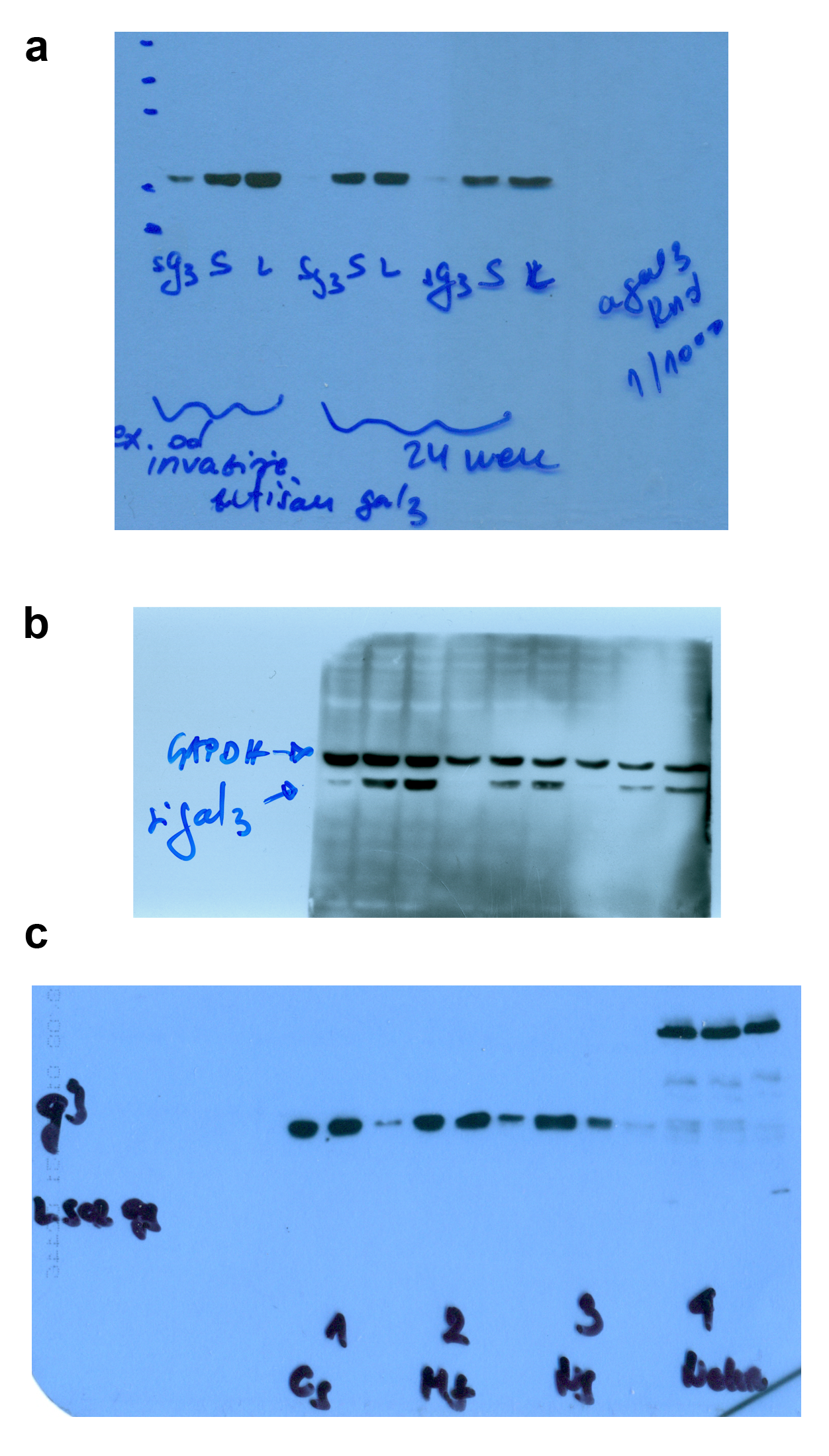
**

**Supplementary Figure S2**

Full-length blots of Figure 4b and 4c. (a) Transfection efficiency of galectin-3 analyzed by Western blot using GAPDH as loading control (b); exposition 3 minutes. (c) Galectin- 3 in cellular compartments after silencing; exposition 7 minutes. The abbreviations for fractions are: Cs or 1-cytoplasmic; Mf or 2-membrane; Ns or 3-nuclear soluble; Nchr or 4-nuclear chromatin. Abbreviations: L-lipofectamine, scr-scrambled siRNA, g3-galectin-3 transfected.

**
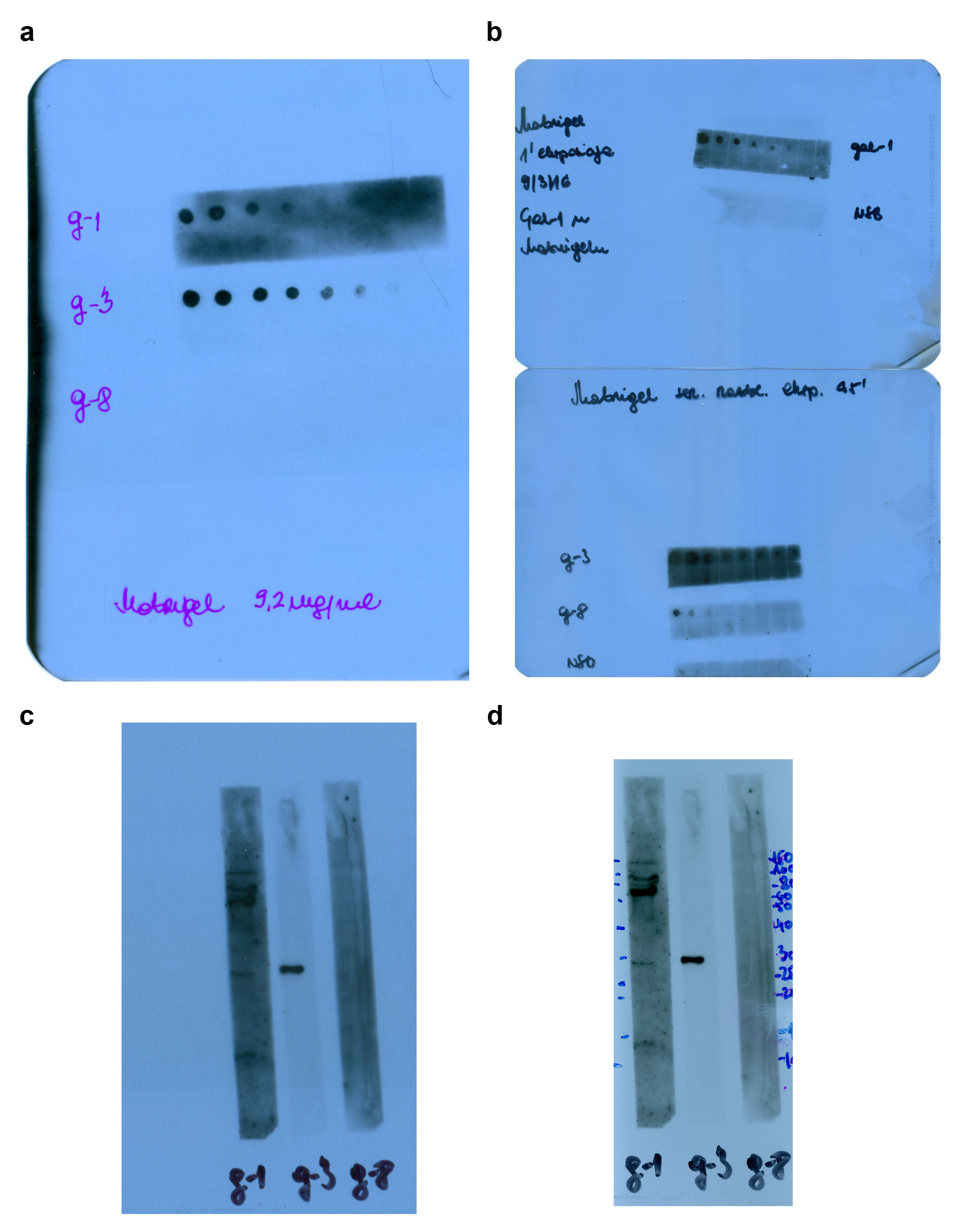
**

**Supplementary Figure S3**

Dot-blot and full-length blots of Figure 5c and 5d. (a, b) Dot-blot analyses of Matrigel preparations. (c, d) Galectins in different Matrigel preparations; exposition 10 minutes. Abbreviations: g-1-galectin-1; g-3-galectin-3; g-8-galectin-8.

**
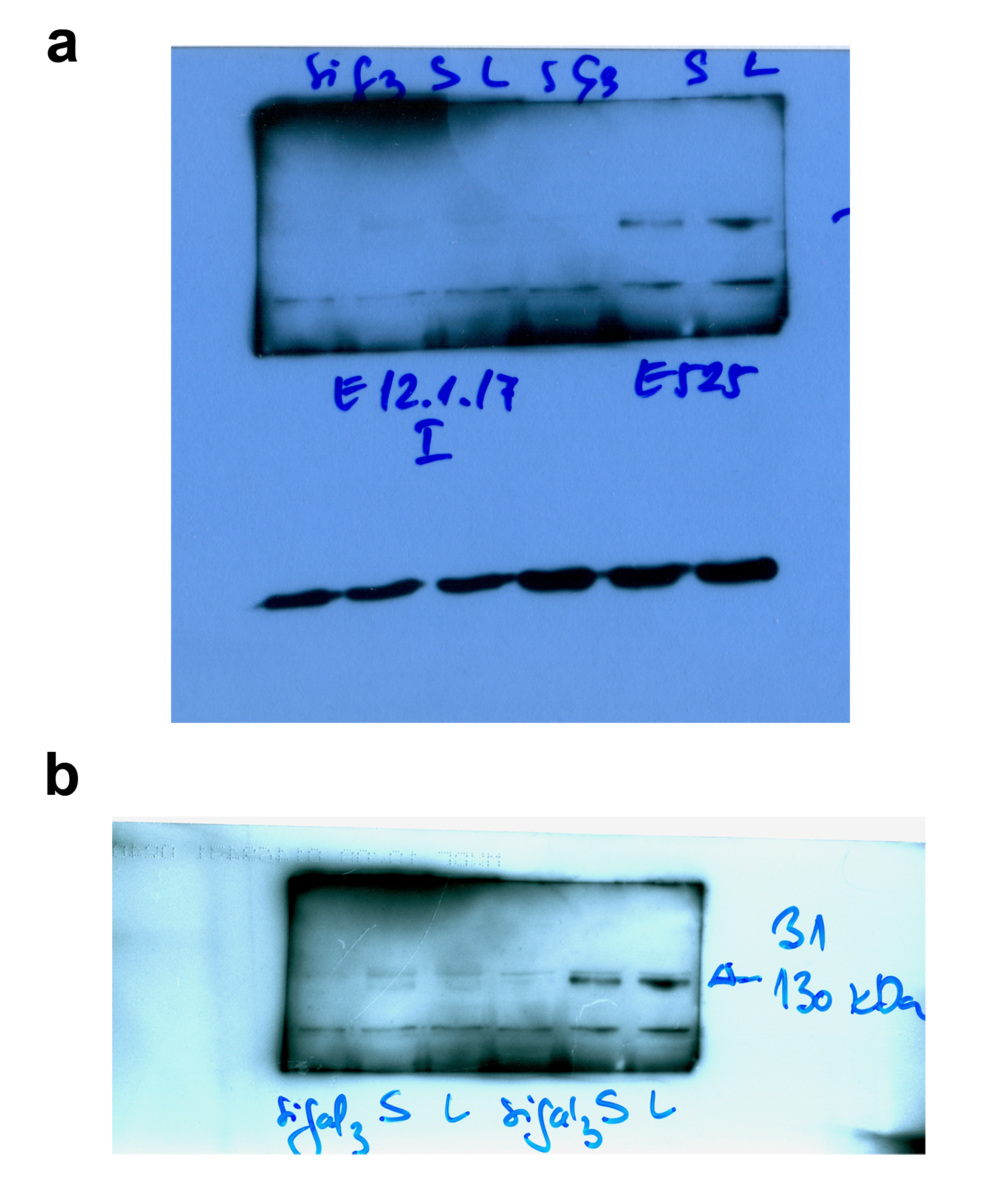
**

**Supplementary Figure S4**

Full-length blot of Figure 6d, 3 individual experiments; exposition 3 minutes. Abbreviations: L-lipofectamine; S-scrambled siRNA; sigal3 galectin-3 transfected; β_1_- integrin β_1_.

**
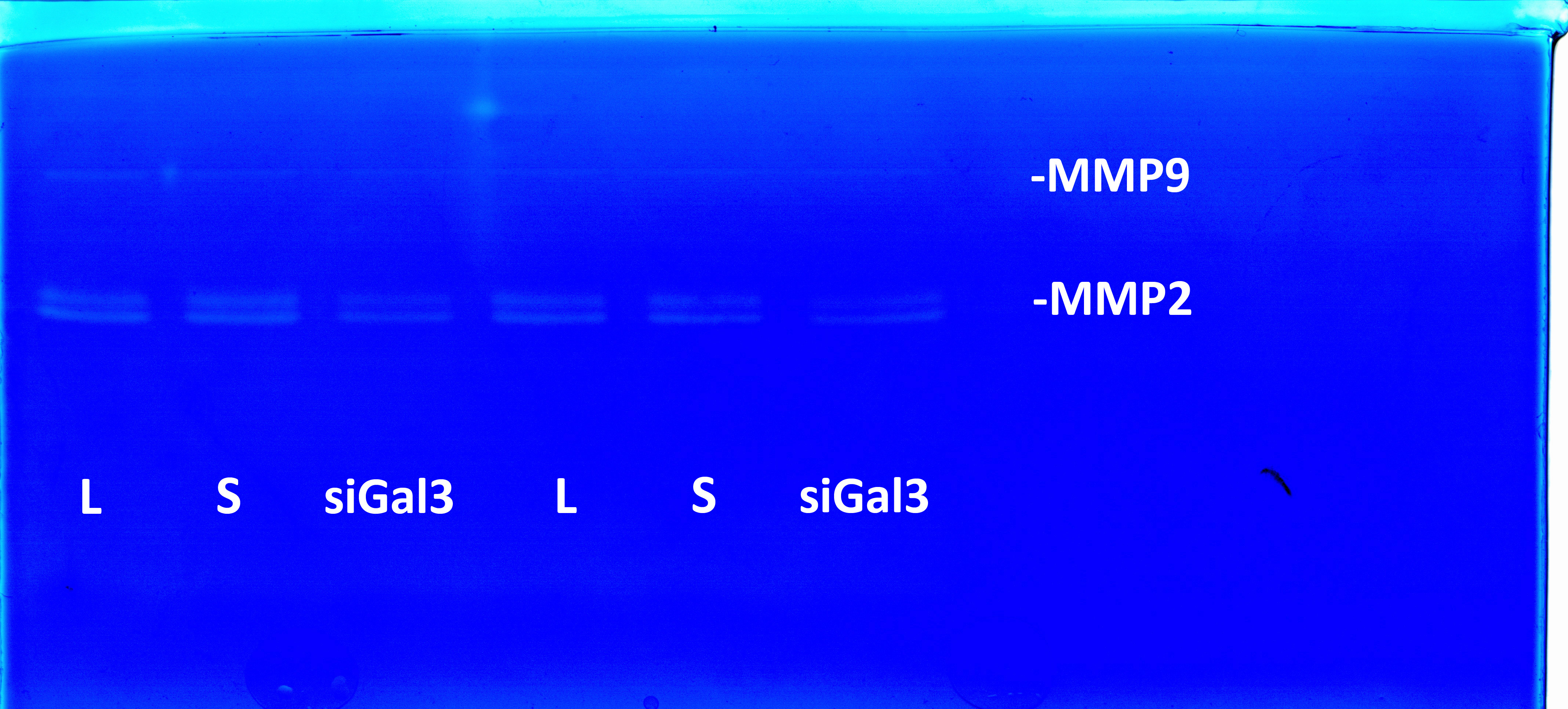
**

**Supplementary Figure S5**

Full-length zymography gel of Figure 7c. Abbreviations: L-lipofectamine; S-scrambled siRNA; sigal3 galectin-3 transfected; MMP2- metalloproteinase 2; MMP9- metalloproteinase 9.


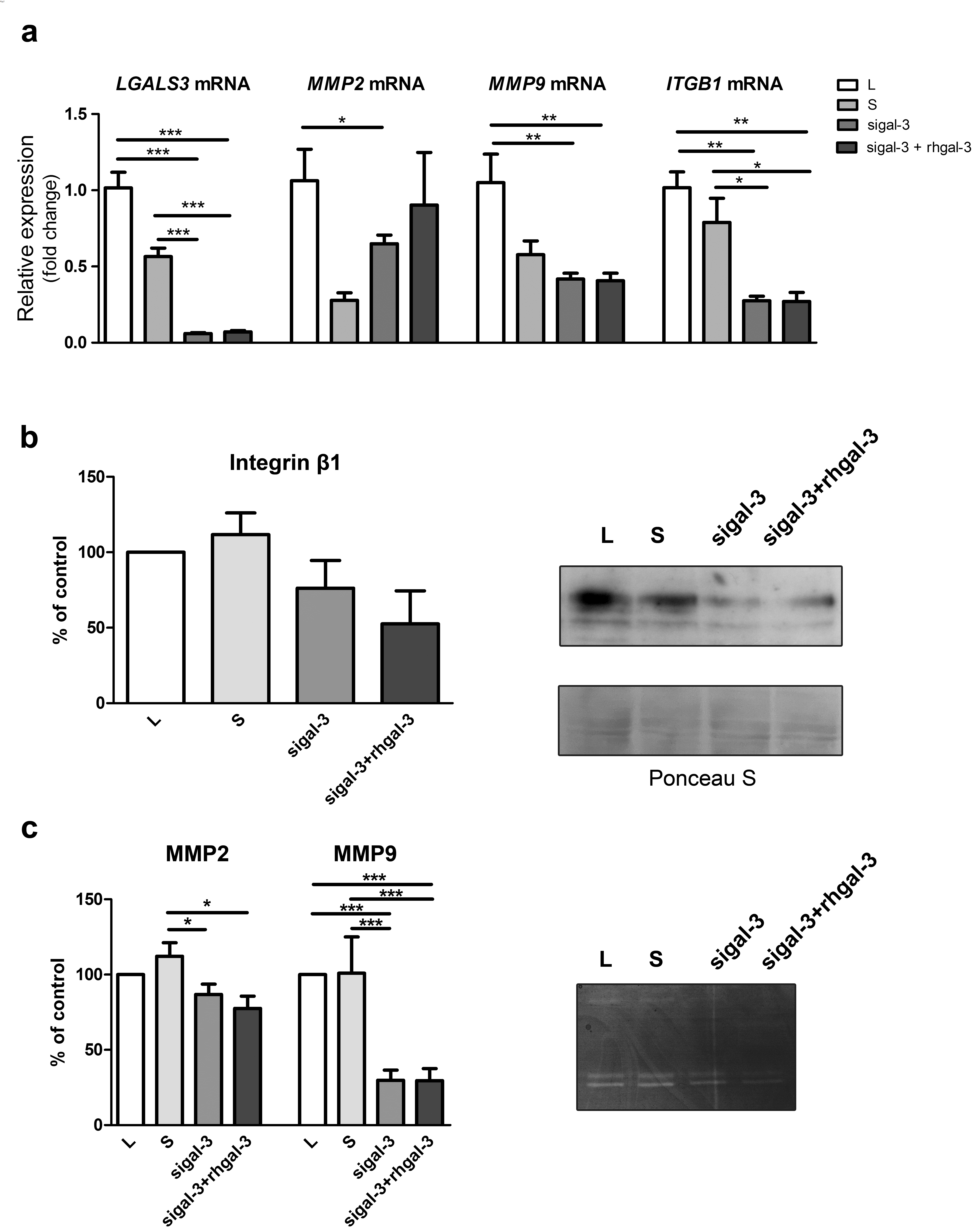


**Supplementary Figure S6**

Rescue experiment (n=3) for sigalectin-3 by extracellular supplementation with rhgalectin-3. No change in mRNA levels of designated genes (a), or protein level of integrinβ_1_ and level/activity of MMP2 and MMP9 were observed. Differences were significant at p<0.05 (*), p<0.01 (**), and at p<0.001 (***).

**
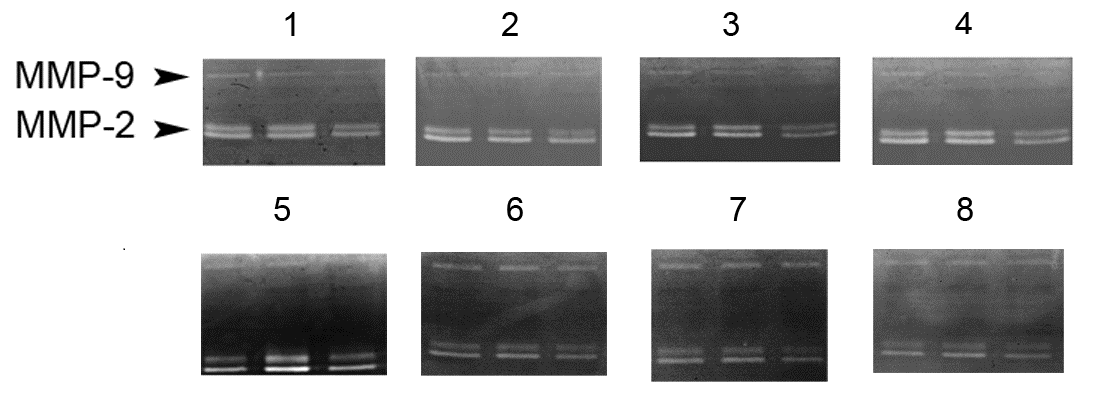
**

**Supplemetary Figure S7**

Zymogrames showing MMP2 and MMP9 from 8 experiments with sigalectin-3 used for statistical evaluation in Fig.7.

**Supplementary Table 1.**

Primers used in real-time PCR

| Gene | Primers |
| --- | --- |
| *LGALS1* | F: TGCAACAGCAAGGACGGC  R: CACCTCTGCAACACTTCCA |
| *LGALS3* | F: CAGAATTGCTTTAGATTTCCAA  R: TTATCCAGCTTTGTATTGCAA |
| *LGALS8* | F: CTTAGGCTGCCATTCGCT  R: AAGCTTTTGGCATTTGCA |
| *MMP2* | F: TGCGACCACAGCCAACTACG  R: ACAGACGGAAGTTCTTGGTGTAGG |
| *MMP9* | F: TGACAGCGACAAGAAGTG  R: CAGTGAAGCGGTACATAGG |
| *ITGB1* | F: GTGGTTGCTGGAATTGTTCTTATT  R: TTTTCCCTCATACTTCGGATTGAC |
| *ITGA1* | F: GGTTCCTACTTTGGCAGTATT  R: AACCTTGTCTGATTGAGAGCA |
| *ITGA5* | F: GGCAGCTATGGCGTCCCACTGTGG  R: GGCATCAGAGGTGGCTGGAGGCTT |
| *GAPDH* | F: GAAGGTGAAGGTCGGAGT  R: GAAGATGGTGATGGGATTTC |
